# CD317 functions as a key antiviral factor in human herpesvirus 6 (HHV-6) infection

**DOI:** 10.1128/jvi.00841-25

**Published:** 2025-06-24

**Authors:** Xianyi Xu, Minmin Song, Xin Zhang, Junli Jia, Shuhua Chen, Hua Xie, Lingyun Li, Jingjing Ma, Huamin Tang

**Affiliations:** 1 Department of Immunology, National Vaccine Innovation Platform, School of Basic Medical Sciences, Nanjing Medical University 12461, Nanjing, China; 2 Department of Critical Care Medicine, Changzhou Cancer Hospital 162737, Changzhou, China; 3 Jintan Hospital Affiliated to Jiangsu University, Changzhou, China; 4 Department of Medical Genetics, Nanjing Medical University 12461, Nanjing, China; 5 Department of Clinical Laboratory, Children’s Hospital of Nanjing Medical University, Nanjing, China; 6 The Laboratory Center for Basic Medical Sciences, Nanjing Medical University 12461, Nanjing, China; University of Virginia, Charlottesville, Virginia, USA

**Keywords:** CD317, HHV-6, gO

## Abstract

CD317, an interferon-stimulated gene, is known for its role in inhibiting the release of various enveloped viruses from infected cells. However, its function can vary, as it also promotes infection in certain contexts, such as with human cytomegalovirus (HCMV). Human herpesvirus 6 (HHV-6) and HCMV are both classified within the β-herpesvirus subfamily. The role of CD317 in HHV-6 infection has not been previously investigated. In this study, we found that (i) HHV-6 infection induces CD317 expression, which in turn restricts HHV-6 infection, (ii) type I interferon stimulation induces CD317 expression, thereby inhibiting HHV-6 infection, (iii) the HHV-6 envelope glycoprotein O (gO) interacts with CD317, leading to gO degradation, and (iv) CD317 is incorporated into HHV-6 virions. This work represents the first report elucidating the role of CD317 in HHV-6 infection and reveals a novel function of gO in this process.

**IMPORTANCE:**

Upon stimulation with type I interferon, hundreds of interferon-stimulated genes (ISGs) are induced to express. For an individual virus, it is crucial to identify and analyze the key ISGs. Here, we discovered that CD317 is one of the key ISGs that restrict HHV-6 infection. While CD317 is well known for its ability to inhibit the release of progeny virions, we have revealed a novel role for CD317 in restricting HHV-6 infection by inhibiting viral entry. Additionally, we found that CD317 interacts with HHV-6 glycoprotein O (gO), a protein of unknown function, leading to the proteasomal degradation of gO. This finding may provide valuable clues for further analysis of gO’s function.

## INTRODUCTION

The Herpesviridae family consists of a diverse group of enveloped DNA viruses, classified into three subfamilies: α, β, and γ. Human herpesvirus 6 (HHV-6), human cytomegalovirus (HCMV), and human herpesvirus 7 belong to the β-herpesvirus subfamily. HHV-6 was first identified in 1986 from patients with lymphoproliferative disorders (1) and was initially classified into two variants, HHV-6A and HHV-6B, based on genetic and tropism differences (2–4). These variants were later reclassified as distinct species in the 2012 Virus Taxonomy List (5). HHV-6 is the causative agent of exanthem subitum (roseola infantum) during primary infection. The virus typically infects most infants between 6 and 12 months of age, establishing lifelong latency. Reactivation of HHV-6 in immunosuppressed or immunocompromised individuals can lead to severe complications and diseases (6–8).

CD317, also known as BST-2, tetherin, or HM1.24, is a type II transmembrane protein (9–12) belonging to the interferon-stimulated gene (ISG) family (13, 14). ISGs are crucial components of the host’s antiviral defense, activated by the type I interferon (IFN-I) signaling pathway, which regulates the host immune response (13, 15–17). CD317 exhibits antiviral properties, including the ability to restrict the release of virions from infected cells (18–25) by tethering them to the plasma membrane. Its structural features include a cytoplasmic domain, a single membrane-spanning α-helix, an extracellular domain, and a C-terminal glycosylphosphatidylinositol anchor (26). Human CD317 is glycosylated and possesses a molecular mass of 30–36 kDa. Through these unique features, CD317 directly inhibits the release of various viruses, such as HIV-1, HIV-2, SIV, KSHV, HSV-1, HSV-2, and SARS-CoV-2 (21, 27–31).

Two mechanisms have been proposed for CD317’s antiviral activity: the “membrane-spanning model” and the “extracellular (EC) self-interaction model.” In the membrane-spanning model, CD317 prevents viral release by tethering virions on the plasma membrane. The EC self-interaction model suggests that CD317 monomers may interact between the cell and viral membranes (32), creating a physical bridge that could influence viral entry or release. Given these models, CD317 plays a multifaceted role in viral infections, either restricting or promoting viral processes depending on the virus and context (18, 19, 22, 33–38).

While CD317’s antiviral role is well documented in herpesvirus infections, its effects are virus specific. For example, CD317 restricts HSV-1, HSV-2, and KSHV release (10, 21, 29, 39) yet enhances HCMV infection at the entry stage (40). This suggests a complex, context-dependent role for CD317 in herpesvirus infections. Despite the known interplay between CD317 and various viral glycoproteins (10, 21, 25, 29, 37, 39, 41–43), the function of CD317 in HHV-6 infection has remained unclear. In this study, we aimed to investigate the role of CD317 in HHV-6 infection. Our findings indicate that CD317 acts as a restriction factor and is incorporated into the HHV-6 virion. Moreover, we identified a novel interaction between CD317 and the HHV-6 glycoprotein O (gO), which may provide new insights into the mechanisms of HHV-6 pathogenesis.

## MATERIALS AND METHODS

### Cell lines and virus cultures

MT4, Molt3, JJhan, and HSB-2 cells were cultured in RPMI 1640 medium (Gibco) supplemented with 10% fetal bovine serum (FBS; Gibco). The human cervical epithelial cell line HeLa, human embryonic kidney cell line 293T, and African green monkey kidney cell line Vero were maintained in Dulbecco’s modified Eagle medium (Gibco), also supplemented with 10% FBS. Umbilical cord blood mononuclear cells (CBMCs) were isolated by Ficoll gradient centrifugation (Tianjin Haoyang Biological Manufacture Co., Ltd.) and cultured in RPMI 1640 medium with 10% FBS, phytohemagglutinin (40 mg/mL; MultiSciences Biotech Co., Ltd.), and interleukin-2 (2 ng/mL; Sigma). The HHV-6 strain Z29 was propagated in CBMCs as previously described (44).

### Plasmid construction

HHV-6 IE1 plasmids for antigen protein expression were constructed as previously described (45, 46). The gene sequences for HA-tagged gO, gB, gQ1, gQ2, gH, gL, gM, and gN expression have been described previously (47–50). Additionally, the gp96 gene sequence was cloned into the pCAGGS plasmid for antigen protein expression (51). The HA-tagged ubiquitin plasmids were kindly gifted by Dr. Yang’s Lab at Nanjing Medical University (52).

### Antibodies

A rabbit polyclonal antibody against human CD317 was kindly provided by the National Institutes of Health (NIH). Monoclonal antibodies for Flag (F1804) and HA (H-9658) tags were purchased from Sigma. Mouse monoclonal antibodies against β-actin and HRP-conjugated goat anti-mouse IgG (H + L) were obtained from Proteintech. HRP-conjugated mouse anti-rabbit IgG (H + L) was sourced from Santa Cruz Biotechnology. An in-house mouse monoclonal antibody against gp96 was also used (51). UV-inactivated HHV-6 virions (Z29 strain) were used as an antigen to immunize mice, generating anti-gQ1, anti-gH, anti-gL, and anti-gO antibodies (44). Recombinant HHV-6B IE1 proteins expressed in *E. coli* (BL21 strain) were used for raising antibodies against the HHV-6B IE1 protein.

### IFN-β stimulation assay

CBMCs, MT4, and Molt3 cells (1 × 10^6^ cells per sample) were cultured in RPMI 1640 medium supplemented with 10% FBS in a 12-well plate. Cells were stimulated with IFN-β (100 U/mL; Genscript) for 24 or 48 hours, then collected and analyzed by SDS-PAGE and Western blotting.

### Western blotting analysis

Cells were collected and lysed using radioimmunoprecipitation assay buffer (Beyotime). Lysates were separated by SDS-PAGE and transferred onto polyvinylidene difluoride membranes. Membranes were incubated with primary antibodies followed by horseradish peroxidase (HRP)-conjugated secondary antibodies. Bands were visualized using enhanced chemiluminescence reagents (Tanon).

### Generation of CD317 overexpressing and knockdown cells

CD317 with a Flag tag at residue 146 (CD317-Flag) was generated by PCR amplification and inserted into the lentiviral vector CSCA-MCS to create CSCA-CD317-Flag as described previously (29). Lentiviruses expressing CD317Flag were generated by transfecting 293T cells with pCAG-HIVgp, pCMV-VSV-G-RSV-Rev, and CSCA-CD317-Flag plasmids. For the knockdown experiment, the pLKO.1 plasmid was modified to include EcoRI (TaKaRa) and AgeI (TaKaRa) restriction sites for oligonucleotide insertion. Briefly, the plasmid was amplified using the primers 5′-ACAGAATTCACCGGTGTTTCGTCCTTTC-3′ and 5′-ACAGAATTCTCGACCTCGAG-3′, followed by digestion with EcoRI and self-ligation of the PCR product. The resulting plasmid contained EcoRI and AgeI sites for subsequent cloning of shRNA sequences.

The target sequences for CD317 knockdown were 5′-AGGGAGAGATCACTACATTAA-3′, 5′-TGCTCCTGATCATCGTGATTC-3′, and 5′- GTGGGAATCGTGGATAAGAAGTA-3′, and the control sequence was 5′-CAACAAGATGAAGAGCACCAA-3.′ The sequences were cloned into the AgeI and EcoRI sites of the modified pLKO.1 vector.

For CD317 knockdown, the modified pLKO.1 lentiviral vector containing the shRNA-CD317 construct was transfected into 293T cells along with packaging plasmids. The lentiviruses were harvested after 48 hours and used to transduce target cells. The cells were selected by puromycin treatment, and CD317 expression was assessed by Western blot.

### Quantitative PCR

T cells overexpressing or knocked down for CD317 were infected with HHV-6 or mock-infected. At 72 hours post-infection (hpi), 200 µL of the cell solution was collected for virus quantification. Cell-associated virus (CV) and supernatant virus (SV) were harvested, and total DNA was extracted using phenol-chloroform extraction. For quantitative PCR, primers specific for the U31 region of the HHV-6 genome (forward: 5′-TTTGCAGTCATCACGATCGG-3′; reverse: 5′-AGAGCGACAAATTG GAGGTTTC-3′) were used, with GAPDH primers (forward: 5′-GGGAAG CTCACTGGCATGG-3′; reverse: 5′-TTACTCCTTGGAGGCCATGT-3′) as an internal control. Quantitative PCR (qPCR) was performed on an ABI StepOnePlus system, using the following conditions: 95°C for 1 minute, followed by 40 cycles of 95°C for 15 s, 60°C for 15 s, and 72°C for 45 s. Gene expression was analyzed using the 2^-ΔΔCt^ method.

### Viral adsorption assay

For viral adsorption, cells were pre-cooled to 4°C for 10 minutes, centrifuged at 800 × *g* to remove the medium, and then incubated with HHV-6 for 1 hour at 4°C with gentle rotation. Cells were washed with cold PBS and collected. Viral DNA was extracted and quantified by qPCR.

### Viral invasion assay

For the viral invasion assay, cells were pre-cooled to 4°C for 10 minutes, centrifuged at 800 × *g* to remove the medium, and incubated with HHV-6 for 1 hour at 4°C. Cells were washed with cold PBS, then incubated at 37°C for 2 hours in medium with 2% FBS. After three washes with cold citric buffer (50 mM sodium citrate and 4 mM KCl, pH 3.0) (53), cells were collected and analyzed by qPCR.

### Purification of HHV-6 virions

Supernatants from HHV-6-infected CBMCs were collected and centrifuged at 2,000 × *g* for 10 minutes at 4°C. The supernatant was filtered through a 0.45 µm filter, layered over a 20% sucrose solution, and subjected to ultracentrifugation at 100,000 × *g* for 1 hour at 4°C. The virion pellet was resuspended with culture medium and stored for further analysis.

### Immunoelectron microscopy

Immunoelectron microscopy of purified HHV-6 virions was performed as previously described (54). Following immunogold labeling, samples were examined using a FEI Tecnai G2 transmission electron microscope.

### Co-immunoprecipitation

Antibodies were conjugated to protein G-Sepharose (GE Healthcare) and cross-linked with dimethyl pimelimidate (Thermo Scientific) as per the manufacturer’s instructions. Cells were lysed with TNE buffer (10 mM Tris-HCl [pH 7.8], 0.15 M NaCl, 1 mM EDTA, and 1% NP-40) containing a protease inhibitor cocktail (Sigma-Aldrich). After centrifugation at 13,000 × *g* for 1 hour at 4°C, the supernatant was incubated with antibody-bound protein G-Sepharose overnight at 4°C. Immunoprecipitated proteins were eluted with 0.1 M glycine (pH 2.8), neutralized, and analyzed by Western blotting.

### Immunofluorescence assay

HeLa cells were transfected with gO- and CD317-expressing plasmids. After fixation, the cells were incubated with primary antibodies (anti-gO and anti-CD317), followed by incubation with appropriate secondary antibodies. After washing, cells were mounted with coverslips and imaged using the Zeiss LSM880NLO confocal microscope.

### Statistical analysis

Data are expressed as the mean ± standard deviation (SD). All experiments were performed at least three times. Statistical significance was determined using unpaired *t*-tests. *P*-values less than 0.05 were considered statistically significant. Densitometric analysis of Western blot bands was carried out using ImageJ (NIH, Bethesda, MD, USA).

## RESULTS

### HHV-6 infection induces CD317 expression in host cells

**Fig 1:**
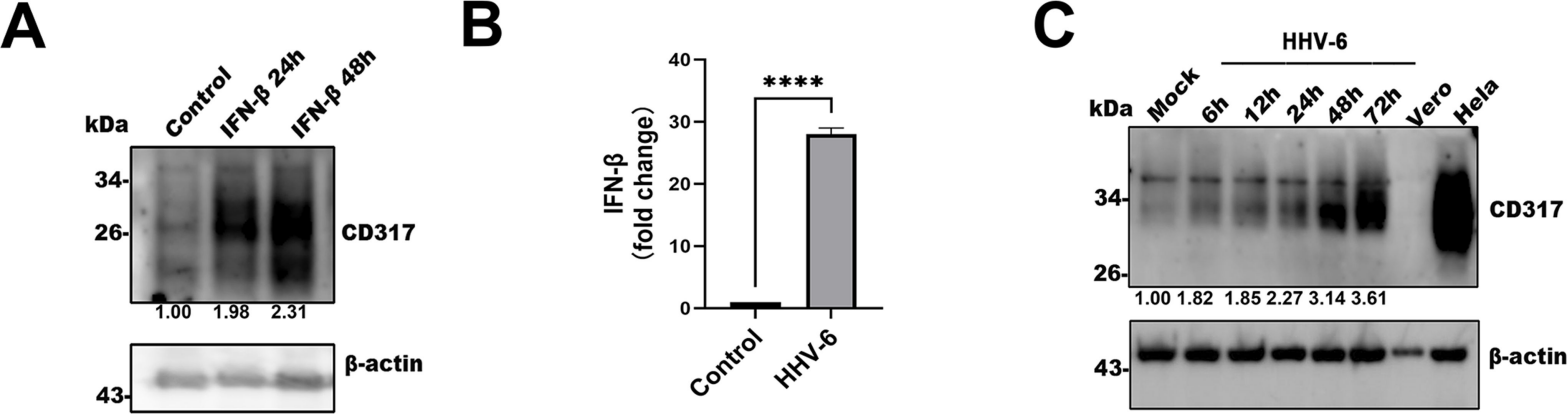
HHV-6 infection induces CD317 expression in host cells. (**A**) Effect of IFN-β stimulation on CD317 expression. Western blot analysis of CD317 expression in untreated and IFN-β-treated MT4 cells at 24 and 48 hours post-treatment. β-actin was used as an internal control. (**B**) IFN-β expression in control and HHV-6-infected cells. HHV-6-infected MT4 cells were harvested at 72 hpi, and IFN-β mRNA expression was quantified by RT-qPCR, normalized to β-actin expression. (**C**) Expression of CD317 in mock and HHV-6-infected T cells. MT4 cells were infected with HHV-6 or mock-treated, and CD317 levels were measured by Western blotting at 6, 12, 24, 48, and 72 hours post-infection. Vero cells served as a negative control, and HeLa cells were used as a positive control.

To investigate the role of CD317 in HHV-6 infection, we first assessed whether CD317 expression could be induced by type I interferon. As shown in Fig. 1A, IFN-β stimulation for 24 and 48 hours significantly increased CD317 levels in Molt3 cells, consistent with its classification as an interferon-stimulated gene. Subsequently, we evaluated whether HHV-6 infection could also induce IFN-I expression in susceptible MT4 cells. Upon infection, IFN-I expression was significantly upregulated, with a progressive increase observed throughout the course of infection (Fig. 1B). Importantly, the increased expression of CD317 was found to correlate with the duration of infection. This finding further supports the notion that CD317 is induced as a response to viral challenge (Fig. 1C).

### CD317 inhibits HHV-6 infection

**Fig 2:**
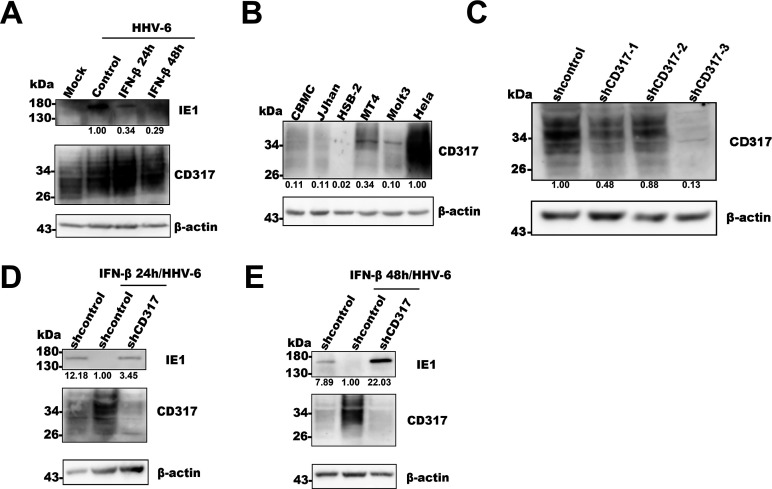
CD317 inhibits HHV-6 infection. (**A**) Expression of HHV-6 IE1 upon IFN-I stimulation. MT4 cells were stimulated with IFN-I for 24 and 48 hours, followed by HHV-6 infection for 24 hpi. Western blotting was performed to detect the expression of the HHV-6 IE1 protein in control and experimental groups. β-actin served as the internal control. (**B**) Endogenous CD317 expression analysis in host cells. Western blotting was used to detect endogenous CD317 in HHV-6-susceptible T cell lines (JJhan, HSB-2, MT4, and Molt3) and CBMCs. HeLa cells were used as a positive control. (**C**) Knockdown efficiency of CD317-targeting shRNAs. MT4 cells were transduced with the lentiviruses expressing control- or CD317-targeting shRNAs for 48 hours. Western blotting was used to assess CD317 expression, with β-actin as an internal control. (**D, E**) Expression of HHV-6 IE1 upon IFN-I stimulation after CD317 knockdown. MT4 cells were transduced with lentivirus for CD317 knockdown and stimulated with IFN-I for 24 and 48 hours. HHV-6 infection was performed after stimulation, and the expression of IE1 was analyzed by Western blotting. β-actin was used as an internal control.

Given that CD317 is an ISG (13, 14), we hypothesized that its induction by IFN-I would restrict HHV-6 infection. To test this, we pre-stimulated MT4 cells with IFN-β for 24 or 48 hours, followed by HHV-6 infection. The expression of a viral immediate early protein IE1 was assessed as a marker of viral infection. As shown in Fig. 2A, IFN-β stimulation significantly reduced IE1 expression in infected cells, suggesting that IFN-I-induced ISGs inhibit viral replication.

To verify that CD317 was the key factor mediating this restriction, we knocked down its expression in T cells. We initially assessed endogenous CD317 expression across several HHV-6-susceptible T-cell lines and CBMCs and found that MT4 cells exhibited relatively higher levels of CD317 expression, with HeLa cells serving as a positive control (Fig. 2B). Therefore, MT4 cells were selected for subsequent knockdown experiments. Three different shRNAs targeting CD317 were designed and evaluated for their knockdown efficiency; among these, shCD317-3 exhibited the highest efficacy (Fig. 2C) and was used for further analyses. As expected, the knockdown of CD317 reversed the inhibitory effect of IFN-I on HHV-6 infection. In these CD317 knockdown cells, IE1 expression was significantly rescued compared with the control (Fig. 2D and E). These results confirm that CD317 plays a crucial role in limiting HHV-6 infection and that its expression is essential for the antiviral effects of IFN-I.

### Overexpression of CD317 reduces HHV-6 entry into host cells

**Fig 3:**
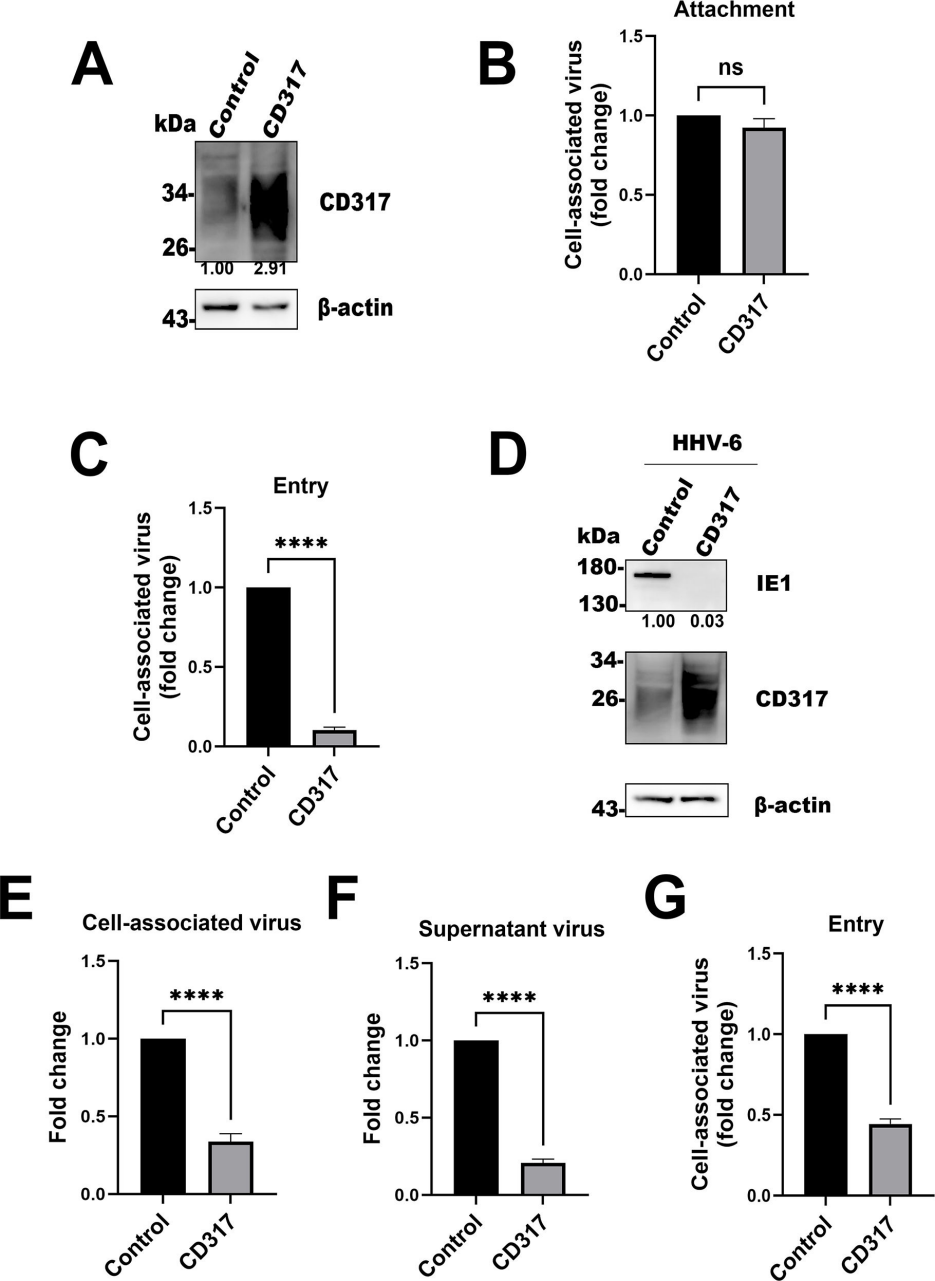
Overexpression of CD317 reduces HHV-6 infection. (**A**) Expression efficiency analysis of CD317. After transducing MT4 cells with control or CD317-expressing lentiviruses for 48 hours, CD317 expression was assessed by Western blotting, using β-actin as an internal control. (**B**) Effect of CD317 overexpression on HHV-6 attachment. MT4 cells overexpressing CD317 or the control cells were incubated with HHV-6 at 4°C for 1 hour, and infection was assessed by qPCR of HHV-6 genome. (**C**) Effect of CD317 overexpression on HHV-6 invasion. After attachment, cells were incubated at 37°C for 2 hours, and infection was analyzed by qPCR. (**D**) CD317 overexpression reduces HHV-6 entry. MT4 cells overexpressing CD317 or the control cells were infected with HHV-6, and expression of HHV-6 IE1 was detected by Western blot at 24 hpi. (**E and F**) CD317 overexpression decreases HHV-6 progeny production. MT4 cells were transduced with the control or CD317-expressing lentiviruses, infected with HHV-6 for 72 hours, and CV or SV levels were quantified by qPCR. Data are presented as fold change over control. Results are mean ± SD of three independent experiments. ns, *P* > 0.5; ****P < 0.01. (**G**) HHV-6 entry analysis using the viruses from CD317-overexpressing and control cells. The viruses from CD317-overexpressing cells and control cells were quantified and were used to infect MT4 cells. Intracellular HHV-6 DNA in target cells was detected via qPCR. Data are presented as fold change over the control. Results are the mean ± SD of three independent experiments. ns, *P* > 0.5; ****P < 0.01.

Next, we sought to directly evaluate the role of CD317 in HHV-6 infection by overexpressing CD317 in T cells. After verifying the efficiency of CD317 overexpression in MT4 cells (Fig. 3A), we proceeded with the subsequent experiments. Cells overexpressing CD317 showed no significant alteration in viral adsorption, as assessed by viral DNA quantification (Fig. 3B). However, overexpression of CD317 significantly reduced viral entry, as demonstrated by reduced levels of viral DNA (Fig. 3C) and decreased expression of IE1 (Fig. 3D). This suggests that CD317 affects the viral entry step of the HHV-6 lifecycle. We further investigated the effects of CD317 overexpression on viral production. At 72 hours post-infection, virus production was measured by qPCR, quantifying both cell-associated virus and supernatant virus. As shown in Fig. 3E and F, both CV and SV were reduced by approximately threefold in CD317-overexpressing cells compared to controls. Finally, we performed an entry assay in MT4 cells using HHV-6 viruses from CD317-overexpressing cells and control cells. After quantifying the viruses, we infected CBMCs with these viruses at the same multiplicity of infection (MOI) and detected intracellular HHV-6 DNA via qPCR. The results showed that the virus from CD317-overexpression cells showed reduced HHV-6 entry into target cells (Fig. 3G).

### Knockdown of CD317 alone does not significantly affect HHV-6 infection

**Fig 4:**
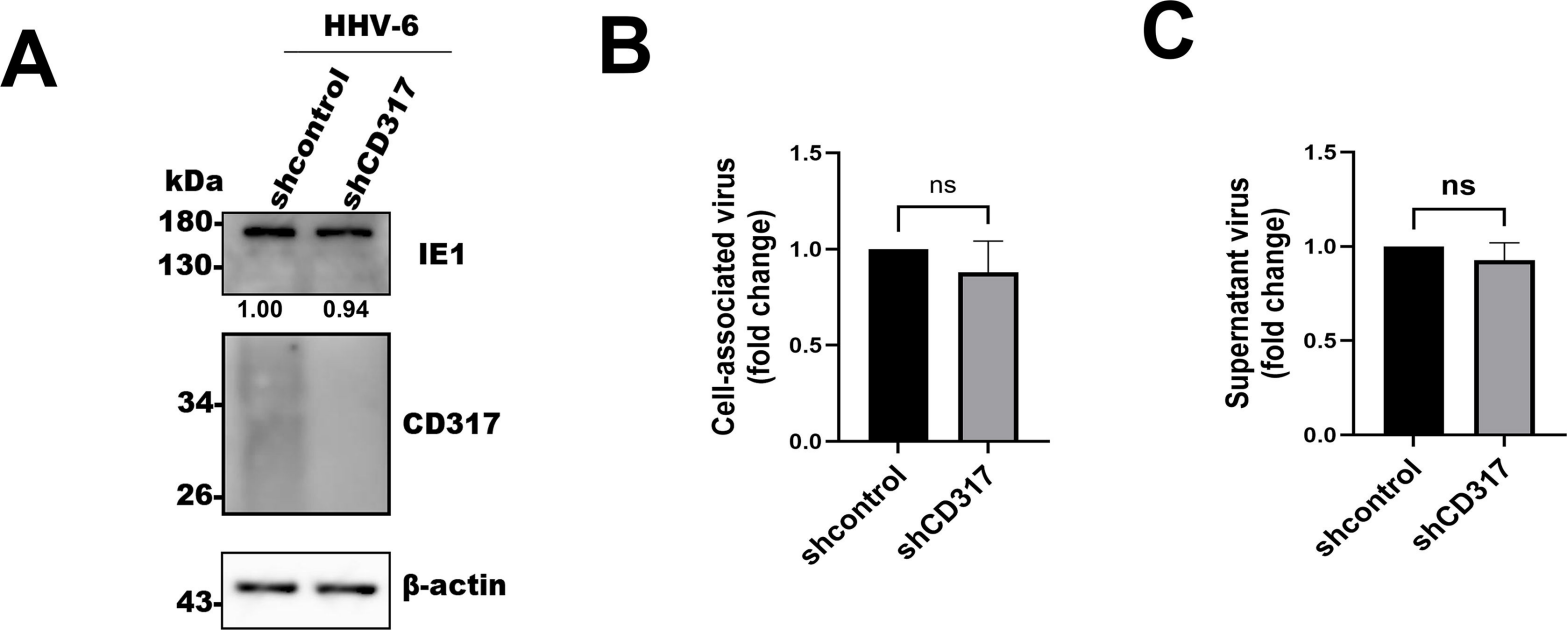
Knockdown of CD317 alone does not significantly affect HHV-6 infection. (**A**) Effect of CD317 knockdown on HHV-6 entry. MT4 cells were transduced with the control or shRNA-CD317 lentiviruses for 48 hours, infected with HHV-6 for 24 hours, and analyzed by Western blotting for IE1 expression. β-actin was used as an internal control. (**B and C**) Effect of CD317 knockdown on HHV-6 progeny production. MT4 cells transduced with control or shRNA-CD317 lentiviruses were infected with HHV-6 for 72 hours, and CV or SV were quantified by qPCR. Data are presented as fold change over control using the 2^-ΔΔCT^ method. Results are mean ± SD of three independent experiments performed in triplicate. ns indicates *P* > 0.5.

To examine the effects of CD317 knockdown on HHV-6 infection, we infected MT4 cells with HHV-6 after silencing CD317 expression via shRNA. Surprisingly, viral infection, as measured by IE1 expression, was similar in CD317 knockdown cells and control cells (Fig. 4A). These results suggest that CD317 may not significantly affect HHV-6 infection in certain cell types or under specific conditions, possibly due to its low baseline expression in MT4 cells.

Additionally, viral production in CD317 knockdown cells was comparable to that in control cells, with no significant difference in either CV or SV levels (Fig. 4B and C). These data suggest that the effect of CD317 on viral infection may be context-dependent, with CD317 overexpression having a more pronounced antiviral effect.

### HHV-6 virions with a low abundance of CD317 demonstrate enhanced efficiency of host cell entry

**Fig 5:**
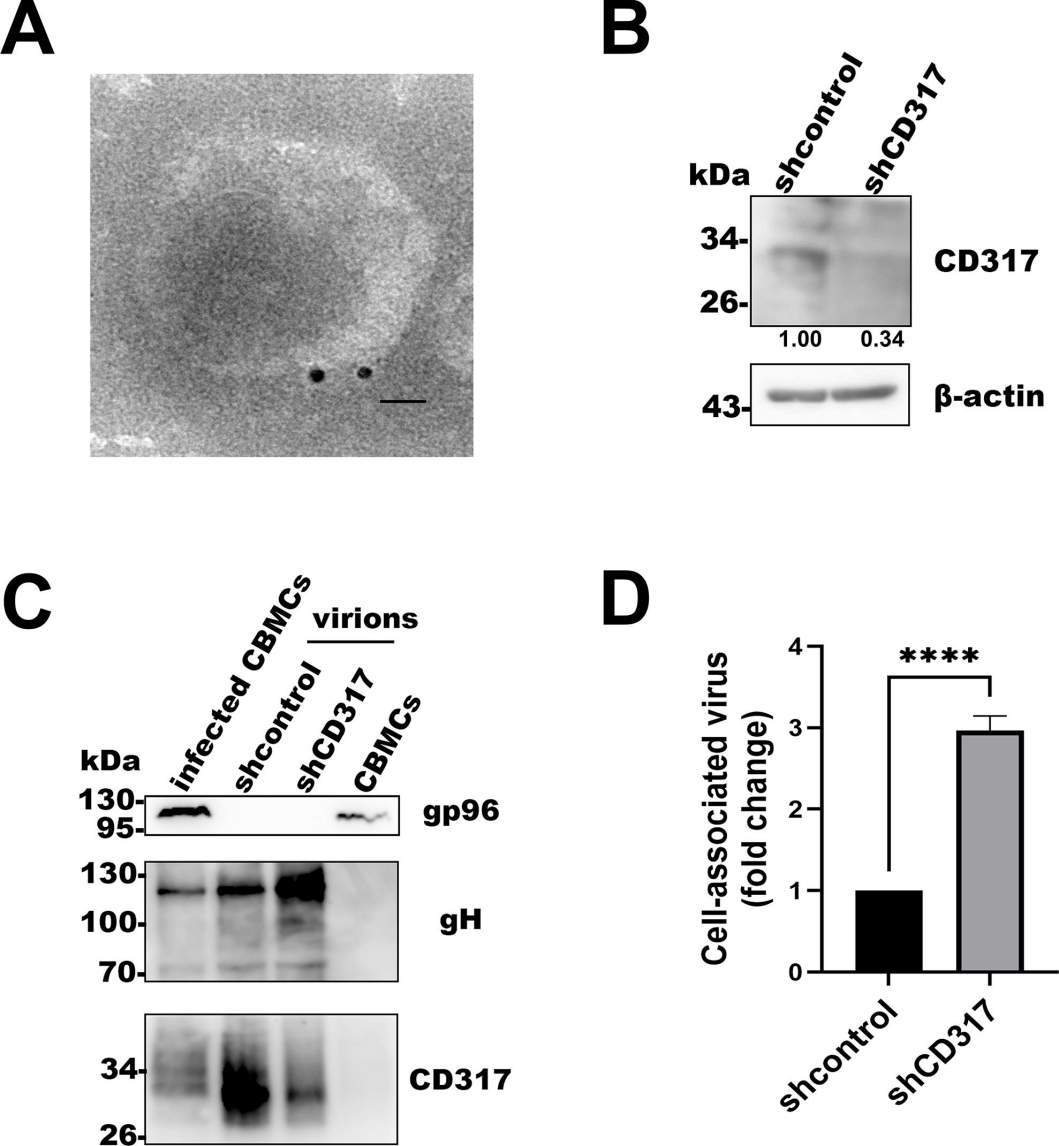
HHV-6 virions with low abundance of CD317 demonstrate enhanced efficiency of host cell entry. (**A**) CD317 is incorporated into HHV-6 virions. Purified HHV-6 virions were fixed, labeled with an anti-CD317 primary antibody, followed by a gold-conjugated secondary antibody, and then visualized using a transmission electron microscope. Scale bar: 25 nm. (**B**) Efficiency of CD317 knockdown in CBMCs. CBMCs were transduced with the lentiviruses for control- or CD317-shRNA expression for 48 hours, and CD317 expression was analyzed by Western blotting, with β-actin as an internal control. (**C**) CD317 detection in HHV-6 virions. HHV-6 was used to infect CD317-knockdown and control cells. After 72 hours, supernatants from these cells were collected, and HHV-6 virions were purified via ultracentrifugation. Western blotting was used to detect CD317 in virions, with gp96 and gH as controls. (**D**) Effect of CD317 in HHV-6 virions on the virus infection. An entry assay was performed on CBMCs using the viruses from CD317-knockdown and control cells. Intracellular HHV-6 DNA was detected via qPCR. Data are presented as fold change over the control. Results are the mean ± SD of three independent experiments. ****P < 0.01.

As there are two models for CD317 to restrict virus infection, we first tested whether CD317 was expressed in HHV-6 virions. HHV-6 virions were purified from infected CBMCs and analyzed by immunoelectron microscopy. As shown in Fig. 5A, CD317 was readily detected in the purified virions. To further investigate the role of virion-associated CD317, we knocked down CD317 expression in CBMCs (Fig. 5B), followed by HHV-6 infection of both knockdown and control cells. Supernatants were collected, and virions were subsequently purified by ultracentrifugation. Western blot analysis confirmed successful virion purification: the cellular marker gp96 was detected only in cells but not in virions, whereas the viral glycoprotein gH was present in both infected cells and virion preparations (Fig. 5C). Notably, virions derived from CD317-knockdown cells contained markedly reduced levels of CD317 compared to those from control cells. These viruses were then quantified by qPCR and used to infect CBMCs at the same MOI. Quantification of intracellular HHV-6 DNA by qPCR revealed that virions produced from CD317-knockdown cells exhibited significantly enhanced entry efficiency (Fig. 5D).

### CD317 mediates degradation of HHV-6 gO partially via the ubiquitin-proteasome pathway

**Fig 6:**
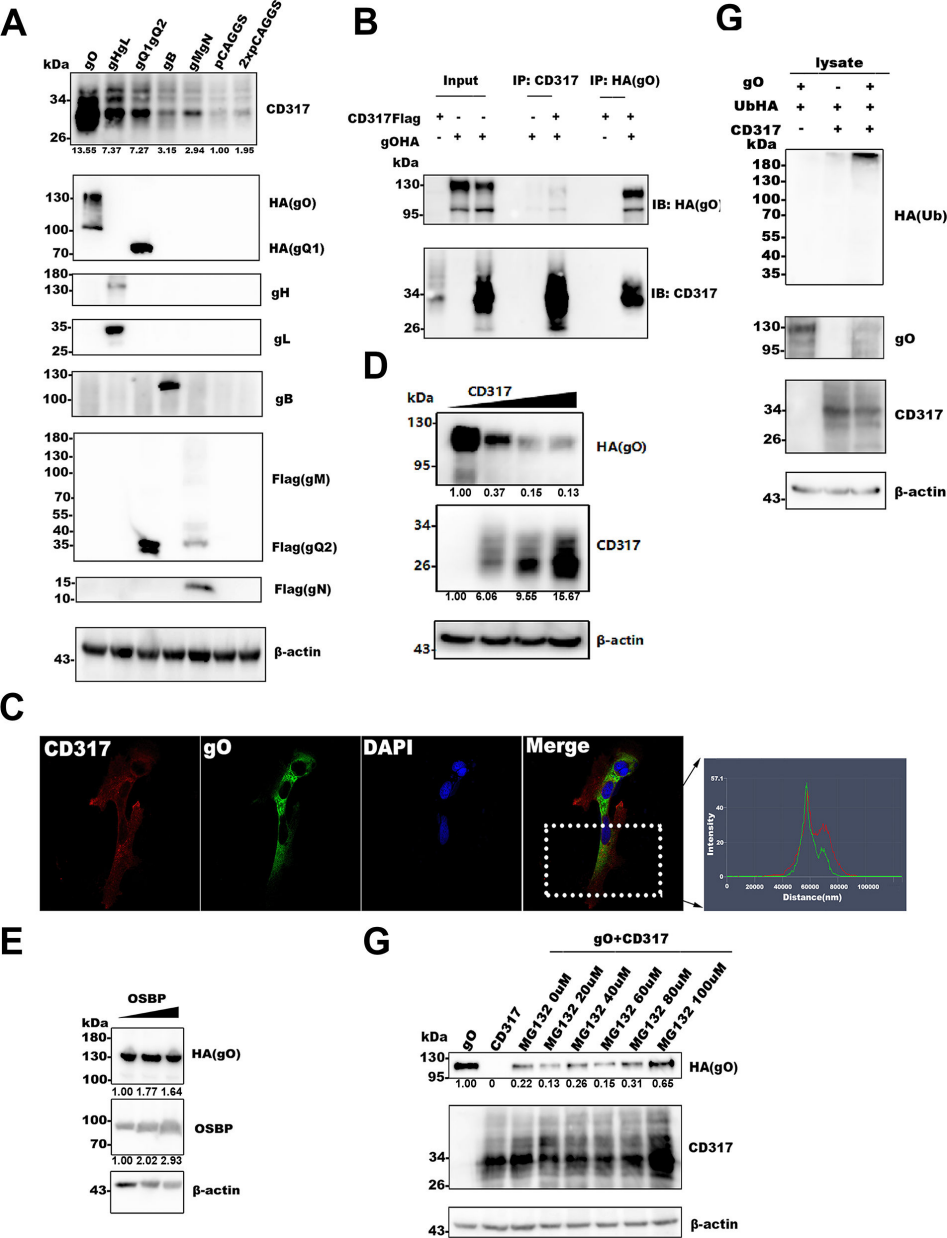
CD317 mediates partial degradation of HHV-6 gO partially via the ubiquitin-proteasome pathway. (**A**) Effect of HHV-6 viral glycoproteins on CD317 expression. 293T cells were co-transfected with the plasmid for CD317 expression and the plasmids expressing HHV-6B gO, gHgL, gQ1gQ2, gB, or gM-gN for 48 hours. (**B**) Co-immunoprecipitation (Co-IP) of CD317 and gO. 293T cells were co-transfected with the plasmids for CD317 and HA-tagged gO expression, followed by Co-IP with anti-CD317 or anti-HA antibodies. Immunoblotting was performed with anti-HA and anti-CD317 antibodies. (**C**) Immunofluorescence validation of the interaction between CD317 and gO. HeLa cells co-transfected with gO- and CD317-expressing plasmids were analyzed by confocal microscope after incubation with primary and secondary antibodies. The image shows CD317 (red), gO (green), and nuclear DAPI (blue) signals in cells. The right panel shows the result of fluorescence intensity distribution analysis. (**D**) CD317 promotes gO degradation. Increasing amounts of CD317 were co-transfected with the gO-expressing plasmid in 293T cells. Western blotting was used to assess the expression of gO and CD317. (**E**) Oxysterol-binding protein (OSBP) does not affect gO expression. 293T cells were co-transfected with varying amounts of OSBP-expressing plasmid together with gO-expressing plasmid. Western blotting was performed to assess the expression levels of gO and OSBP. (**F**) MG132 partially rescues CD317-induced gO degradation. 293T cells co-transfected with CD317- and gO-expressing plasmids were treated with MG132 at varying concentrations for 4 hours before Western blot analysis. (**G**) CD317 promotes gO ubiquitination. 293T cells were co-transfected with gO-, ubiquitin- and CD317-expressing plasmids. Western blotting was performed to assess gO ubiquitination levels.

CD317 restricts viral release effectively through its unique topology, thereby exerting broad-spectrum antiviral activity. However, different viruses can counteract the antiviral activity of CD317 through various viral proteins, such as HIV-1 Vpu, HIV-1 group M Nef, HSV-1 gM, JEV prE, HBV HBx, IAV M2, HSV-2 multiple GP, HIV-2 EnV, KSHV K5, SIV Nef, and Ebola GP. These proteins antagonize the restriction of CD317 through different mechanisms (10, 21, 25, 29, 37, 39, 41–43, 55–57). To investigate the molecular mechanisms underlying the inhibitory effect of CD317 on HHV-6 infection, we explored potential interactions between CD317 and viral glycoproteins. We transfected 293T cells with the plasmids expressing CD317 together with gO, gH/gL, gQ1gQ2, gB, gM, or gN. Western blot analysis revealed a significant increase in CD317 levels in gO-expressing cells (Fig. 6A), indicating a potential association between CD317 and gO.

To further analyze the association, we performed co-immunoprecipitation assays. As shown in Fig. 6B, gO was co-precipitated with CD317. The confocal microscope also confirmed the co-localization of CD317 and gO within the cells (Fig. 6C).

Interestingly, we found that CD317, but not another cellular protein oxysterol-binding protein, led to a significant degradation of gO (Fig. 6D and E). Additionally, CD317-mediated gO degradation could be partially reversed by treatment with the proteasome inhibitor MG132 (Fig. 6F), suggesting that CD317 targets gO for proteasomal degradation. To further address this, 293T cells were co-transfected with gO-, CD317-, and ubiquitin-expressing plasmids to assess gO ubiquitination. The result showed increased gO ubiquitination when it was co-expressed with CD317 (Fig. 6G). Thus, in the present study, although we did not identify an HHV-6 antagonist protein, we may have uncovered a novel mechanism for CD317’s antiviral function—specifically through the degradation of viral glycoproteins.

## DISCUSSION

This study successfully investigates the role of CD317 in human herpesvirus 6 infection. Our findings reveal that CD317 functions as a key restriction factor in the HHV-6 infection process. Notably, CD317 expression is induced not only by IFN-I but also by HHV-6 infection itself. Our data showed that CD317 restricts viral infection by hindering viral entry into host cells and also suggested the role of an interaction between HHV-6 glycoprotein gO and CD317 during this process. Moreover, we show that CD317 is incorporated into viral particles, offering novel insights into the interplay between CD317 and herpesvirus biology.

CD317 is widely recognized as a type I interferon-stimulated gene (13), with its antiviral activity largely attributed to its ability to inhibit the release of viruses from infected cells (e.g., HIV, HSV, and SARS-CoV-2) (18, 19, 21, 22, 24, 27, 39, 58). This study is the first to identify CD317 as a critical restriction factor in the early stages of HHV-6 infection, particularly during viral entry. Overexpression of CD317 in T cells led to a significant reduction in viral invasion, decreased expression of the immediate-early gene IE1, and a marked decrease in viral production, both intracellularly and in the supernatant (Fig. 3). These results extend the antiviral activity of CD317, suggesting it plays a vital antiviral role in the early stage for some viruses.

**Fig 7:**
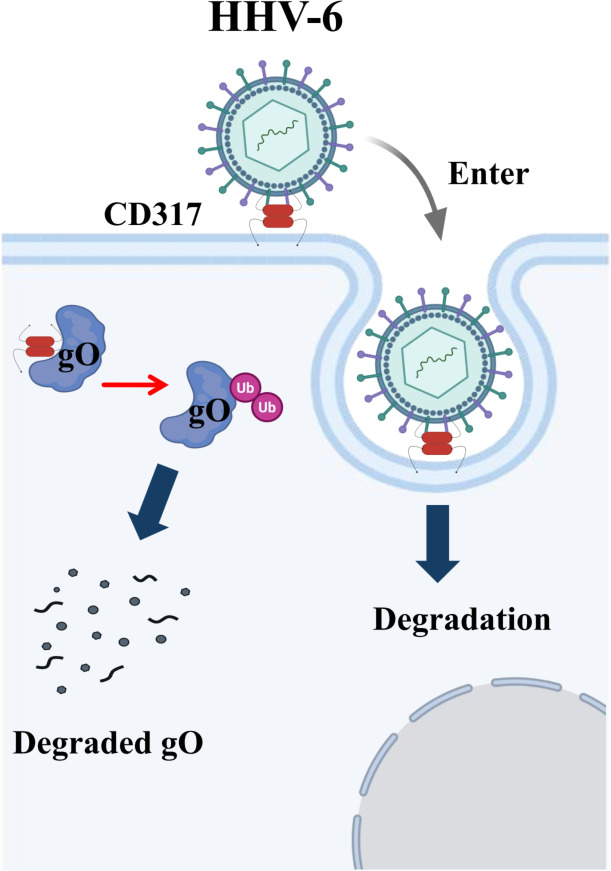
Model of the antiviral mechanism of CD317 during HHV-6 infection. Two possible mechanisms of CD317 antiviral function were suggested in this study: by tethering the incoming viral particles to the cell membrane for degradation, and by interacting with a viral glycoprotein, gO, and promoting its degradation partially through the ubiquitin-proteasome pathway.

CD317’s restriction of HHV-6 entry into cells may be linked to its unique topology. In this study, CD317 was detected in T cells (Fig. 2B) and HHV-6 virions (Fig. 5A). Its extracellular domains are embedded in the host cell and viral membranes, enabling intermolecular interactions through dimerization or trimerization (59). This structure likely tethers the virus to the cell surface, restricting HHV-6 entry. When CD317 abundance decreased in HHV-6 virion, HHV-6 entry efficiency significantly increased (Fig. 5D). This supports the model that CD317 limits HHV-6 invasion by “tethering” viral particles to the cell membrane, restricting their entry into T cells (Fig. 7).

Furthermore, overexpression of CD317 resulted in a substantial reduction in the production of progeny virus in the supernatant (Fig. 3F). However, it remains unclear whether this reduction is due to diminished viral invasion or the tethering and degradation of newly synthesized virus particles, a question that merits further investigation in future studies.

Despite the clear inhibition of HHV-6 infection upon CD317 overexpression, we observed that CD317 gene knockdown, in the absence of IFN stimulation, did not significantly promote viral infection (Fig. 4). This phenomenon may reflect the low baseline expression of CD317 in HHV-6-susceptible cells, and/or other environmental conditions under IFN stimulation may also contribute to CD317 function. In certain cell types or under specific conditions, CD317 may not exert a pronounced antiviral effect, or its function may be masked by other antiviral factors.

Prior research has demonstrated that various viruses antagonize CD317’s antiviral activity through specific viral proteins, such as HIV-1 Vpu, HSV-1 gM, and others (10, 35, 39, 43). These proteins degrade CD317 via distinct mechanisms to overcome its restriction. To explore whether a similar mechanism exists for CD317 agonist HHV-6 infection, we investigated potential relations between CD317 and viral glycoproteins. Notably, co-expression of CD317 resulted in the degradation of HHV-6 glycoprotein O (Fig. 6A through F). This observation suggests that CD317 may restrict HHV-6 infection by promoting the degradation of viral glycoproteins, although the precise role of gO in the HHV-6 life cycle remains to be elucidated.

Previous studies have indicated that human cytomegalovirus, a member of the β-herpesvirus family, requires the trimeric glycoprotein complex gH/gL/gO for infection of endothelial cells, with gO interacting with platelet-derived growth factor receptor α as its cellular receptor (60, 61). However, the cellular receptor for gO in HHV-6 infection remains unidentified. In the present study, we report for the first time that HHV-6 gO directly interacts with CD317 (Fig. 6A through C). This discovery may provide new insights into gO function analysis. Given that gO is incorporated into HHV-6 virions (50), the potential contribution of this interaction to viral entry remains unclear when CD317 is expressed at low or undetectable levels in the virion. Future studies should explore the molecular mechanisms underlying the interaction between gO and CD317 and their roles at various stages of the viral life cycle, laying the foundation for more targeted antiviral therapies.

In summary, this study offers a comprehensive analysis of the role of CD317 in HHV-6 infection, elucidating its antiviral function as a restriction factor and its interaction with viral glycoprotein gO (Fig. 7). These findings expand our understanding of the complex role of CD317 in viral infections and highlight its potential as a therapeutic target. Future research will aim to further elucidate the role of CD317 in other herpesvirus infections and explore its specific interactions with HHV-6 gO, advancing our knowledge of its impact on viral pathogenicity and immune responses.
